# A Privileged Working Memory State and Potential Top-Down Modulation for Faces, Not Scenes

**DOI:** 10.3389/fnhum.2019.00002

**Published:** 2019-01-28

**Authors:** Hai Lin, Wei-ping Li, Synnöve Carlson

**Affiliations:** ^1^Zhongshan School of Medicine, Sun Yat-sen University, Guangzhou, China; ^2^Department of Neurosurgery, Shenzhen Second People’s Hospital, The First Affiliated Hospital of Shenzhen University, Shenzhen, China; ^3^Department of Neuroscience and Biomedical Engineering, Advanced Magnetic Imaging Centre, Aalto NeuroImaging, Aalto University School of Science, Espoo, Finland; ^4^Neuroscience Unit, Department of Physiology, Faculty of Medicine, University of Helsinki, Helsinki, Finland

**Keywords:** top-down modulation, face recognition, FFA, functional connectivity, working memory

## Abstract

Top-down modulation is engaged during multiple stages of working memory (WM), including expectation, encoding, and maintenance. During WM maintenance period, an “incidental cue” can bring one of the two items into a privileged state and make the privileged item be recalled with higher precision, despite being irrelevant to which one to be probed as the target. With regard to the different representational states of WM, it’s unclear whether there is top-down modulation on earth sensory cortical areas. Here, We used this behavioral paradigm of “incidental cue” and event-related fMRI to investigate whether there were a privileged WM state and top-down modulation for complex stimuli including faces and natural scenes. We found that faces, not scenes, could enter into the privileged state with improved accuracy and response time of WM task. Meanwhile, cue-driven baseline activity shifts in fusiform face area (FFA) were identified by univariate analysis in the recognition of privileged faces, compared to that of non-privileged ones. In addition, the functional connectivity between FFA and right inferior frontal junction (IFJ), middle frontal gyrus (MFG), inferior frontal gyrus, right intraparietal sulcus (IPS), right precuneus and supplementary motor area was significantly enhanced, corresponding to the improved WM performance. Moreover, FFA connectivity with IFJ and IPS could predict WM improvements. These findings indicated that privileged WM state and potential top-down modulation existed for faces, but not scenes, during WM maintenance period.

## Introduction

Working memory (WM) is a cognitive system of temporarily holding information available for processing with a limited capacity (Baddeley, 2003). When several items are maintained in WM simultaneously, they can be kept in different representational states (Larocque et al., 2014). If one item is more relevant to the WM task or more likely to be probed than others, it can be brought into a privileged state and be easier to be retrieved (Pertzov et al., 2013). By the introduction of “retro-cue” during the WM maintenance period, different representational states can be manipulated for items in WM (Lepsien et al., 2011; Berryhill et al., 2012). Specifically, a retro-cue will give participants a knowledge or expectation about which items to be relevant to the subsequent probed target. And then the cued item will be recalled with higher precision than other uncued items. Interestingly, no matter whether the retro-cue is valid or not, the benefit always exists for the cued item (Gunseli et al., 2015). The neural underpinnings of the retro-cue effect have been investigated in some fMRI studies (Lepsien and Nobre, 2007; Nelissen et al., 2013). In an event-related fMRI study, a retro-cue informed participants the category information of the probed target in a WM task to remember from two categories of faces and scenes (Lepsien and Nobre, 2007). The improved recall precision of the cued item was accompanied with the increased activity in the category-specific brain region involved in object recognition: fusiform face area (FFA) for faces and parahippocampal place area (PPA) for scenes.

Another tool of manipulating different representational states in WM is presenting items in series, with the last item naturally getting into the privileged state, which is known as the “recency effect” (Allen et al., 2014). The last item is recalled with higher accuracy and shorter response time than all previous items. The recency effect is volatile and susceptible to some attention interference such as presenting irrelevant information (Manohar and Husain, 2016). And its magnitude is dependent on the number of items in all. By fMRI, an increased activity in the inferior temporal cortex was found in the recognition of the last item compared to that of previous items in a words-remembered task (Nee and Jonides, 2008). In addition, Oztekin et al. (2009) further identified a decreased activity in hippocampal along with prioritized memory of the last item. These studies suggested differences in both behavioral WM states and neural representations due to the recency effect.

Similar to the invalid retro-cue, an “incidental cue” could bring one of the two items into a privileged state and make the privileged item be recalled with higher precision, despite being irrelevant to which one to be probed as the target (Zokaei et al., 2014). In a WM experiment by Zokaei et al. (2013) participants were required to remember the motion directions of two groups of dots in two different colors, simultaneously appearing above and below a fixation cross. The incidental cue is the colored fixation cross during maintenance period, the color of which was the same to one group of dots. And Participants were required to answer whether the cued group of dots was above or below the fixation cross right after the appearance of the incidental cue. Although the incidental cue was completely irrelevant to which group of dots to be probed, the direction of the cued group was recalled with higher precision compared to that of the other group. Furthermore, by TMS applied to motion sensitive area MT+ after the incidental cue during maintenance period, the benefit of the cued group was impaired along with the improvement in the retrieval of the uncued group, which provided causal evidence about different representational states in WM. This finding is a bit similar to the phenomenon or experience in our life where the memory in the natural visual world can be incidentally enhanced by some associated information. However, with the effect of incidental cue proved on motion direction as a low-level feature of object, it’s unclear whether the same effect would exist for high-level complex objects such as faces and natural scenes.

Different representational states in WM are accompanied with sensory cortical activity biasing, which is mediated via top-down control (Gazzaley and Nobre, 2012). Top-down modulation on early sensory brain areas, from prefrontal and parietal control regions, influences WM performance during both stimulus-present and stimulus-absent stages of WM tasks, to focus our cognitive resources on goal-relevant information (Gazzaley and Nobre, 2012). During WM encoding period, cortical control regions involved in top-down modulation were investigated in fMRI studies (Gazzaley et al., 2007; Chadick and Gazzaley, 2011). Functional connectivity between left middle frontal gyrus (MFG) and a scene-selective visual region was enhanced when scenes were remembered compared to that when scenes were ignored in an object delayed-response task (Gazzaley et al., 2007). In addition, the strength of this coupling correlated with the magnitude of activated enhancement for relevant stimuli and suppression of irrelevant ones in the scene-selective visual region, which suggested that top-down modulation worked via functional couplings. Similarly, another fMRI study revealed visual cortical areas that selectively processing relevant information were functionally connected with the frontal-parietal network including intraparietal sulcus (IPS), inferior frontal junction (IFJ) and MFG, while those processing irrelevant information were coupled with the default network (Chadick and Gazzaley, 2011). Interestingly, the degree of couplings between visual cortices and default network regions predicted the WM performance. During WM maintenance period, the mechanisms of top-down modulation are similar to that during perception, but possibly with additional regulatory functions (Kuo et al., 2011, 2012). By fMRI, a common set of frontal and parietal control areas are involved in mediating sensory cortical activity for different representational states. In a feature delayed-response task, functional connectivity between frontal and posterior visual areas increased after the effective retro-cue and had a relationship with WM performance (Kuo et al., 2011). Besides, a particular brain area, in ventrolateral prefrontal cortex and around inferior frontal gyrus (IFG) and IFJ, has been implicated in regulating the dynamic of neural representations during WM maintenance period (Kuo et al., 2012). A TMS-fMRI study provided causal evidence for the effect of this area on regulating the level of activity of representations in posterior brain areas to guide perception and action (Higo et al., 2011).

With regard to the effect of incidental cue during WM maintenance period, we supposed top-down modulation played a role in mediating the activity of early sensory areas, which would be investigated in our study. We added an incidental cue in a WM task for two categories of complex objects including faces and natural scenes, to study the privileged WM state and underlying top-down modulation.

## Materials and Methods

### Participants

Eighteen right-handed volunteers (mean age, 27.4 ± 6.6 years; eight females) were recruited from universities with pays. This study was approved by the local ethics committee of our institute and informed consent was obtained from all participants. All participants had normal or corrected-to normal vision and were screened to make sure they had no history of neurological or psychiatric diseases and were not taking any psychotropic medications. All participants were naive regarding the purpose of the study.

### Stimuli

**FIGURE 1:**
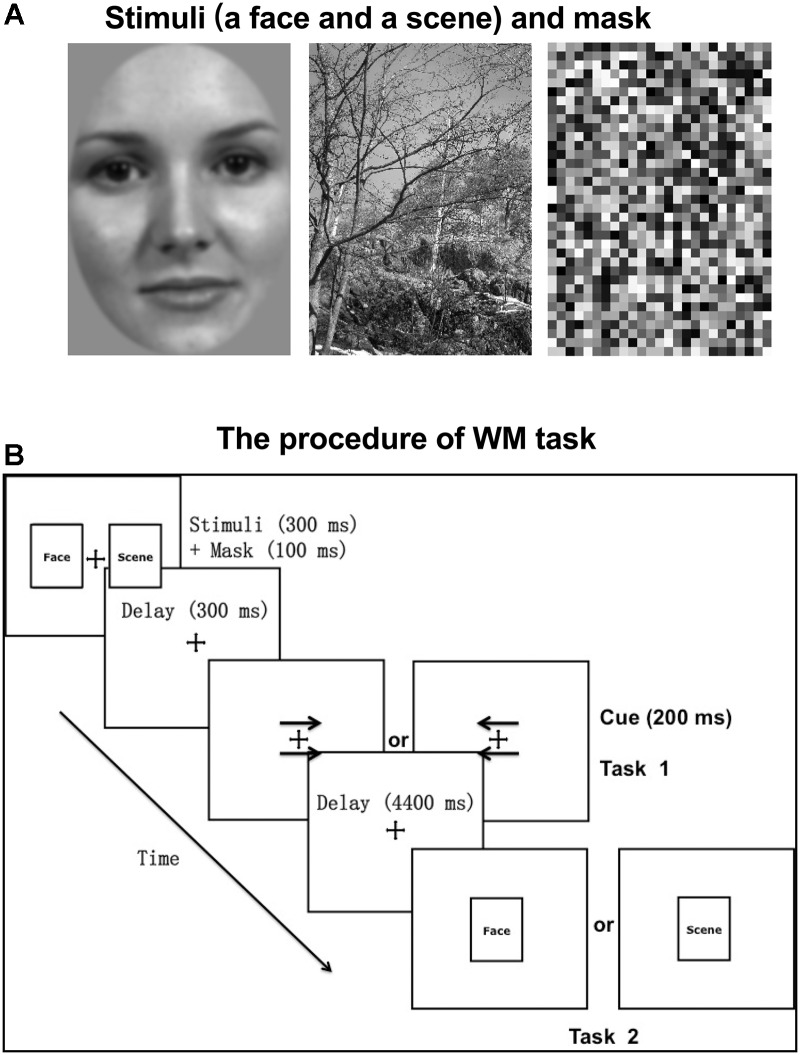
Experimental stimuli and paradigm. **(A)** The stimuli (a face and a scene) and mask presented as an example. Stimuli consisted of grayscale images of 50 neutral faces (half male and half female) on a gray background and 50 natural scenes (3.2° horizontal and 4.5° vertical visual angles, respectively). **(B)** The procedure of WM task. Before the WM task (task 2), participants were required to complete a cue-responding task (task 1). The cue type (arrows pointing to right or left) and category of the probed target (face or scene) were counterbalanced across trials.

Stimuli consisted of grayscale images of 50 neutral faces (half male and half female) on a gray background and 50 natural scenes (3.2° horizontal and 4.5° vertical visual angles, respectively; Figure 1A). Using MATLAB (MathWorks, Natick, MA, United States), all stimuli were grayscale filtered and Gaussian band-pass filtered for spatial frequency with a center spatial frequency of 0.5 cycles/pixel and a Gaussian function sigma value of 0.2 cycles/pixel. The face stimuli were edited so that the main features fit inside an oval window, with the outlines of the stimuli (the edges of the faces) not visible. All images were adjusted to have the same luminous flux.

### WM Task With an Incidental Cue

An event-related fMRI experiment was performed with the behavioral paradigm of incidental cue. The procedure was displayed in Figure 1B. Each trial started with a fixation cross (500 ms), followed by two pictures (one is a face and the other is a scene; 300 ms) and masks (100 ms) on both sides of the fixation cross. The mask consisted of small grids with random gray values and had the same size with stimuli (Figure 1A). The locations of face and scene pictures (left or right) were random across trials. After a 300 ms unfilled delay, two arrows pointing to either the right or the left appeared above and below the fixation cross, which served as the incidental cue. If the arrows pointed to the left, participants were required to recall and answer whether the previous left picture is a face or a scene with a key press. And if the arrows pointed to the right, participants made a response to the previous right picture, correspondingly (Task 1). After a 4400 ms unfilled delay, a face or scene picture (same or different to the previous one) was randomly chosen to appear at the center for 300 ms, regarded as the WM target. Participants were instructed to make a response about whether the target is same or different to the previous picture of the same category, as accurately and soon as possible (Task 2). The target was followed by a 6400 ms intertrial interval during which a blank screen was presented. The category of the picture that the arrows pointed to was irrelevant to the category of the target. That is to say, the incidental cue was uninformative about which category of the picture would be probed. There were 30 trials per block and 6 blocks, separated by a 2-min break.

### Eye Movement Recordings

To ensure that participants’ eyes were fixating on the fixation cross when the two pictures of a face and a scene appeared on both sides of the fixation cross, eye movements were recorded at sampling rate of 1000 Hz with an MRI compatible Eyelink 100 eye-tracker (SR Research, Ottawa, Canada) during scanning. A 9-point calibration and validation were performed before each block of the WM task. The criteria for saccade onset were considered an eye movement velocity of 30°/s and an acceleration of 4000°/s^2^. The trials in which participants didn’t keep the fixation within the 2°× 2° region centered on the fixation cross during the appearance of the two pictures would be discarded, to prevent participants from spontaneously making saccade to one of two pictures with better perception of one picture than the other one.

### Localizer Task

An independent functional localizer task was performed to identify the face-selective region of FFA and the scene-selective region of PPA for each participant. The localizer scan consisted of 2 blocks of fixation (rest), viewing faces and viewing scenes (task), respectively, with the duration of 20 s for each block. Each task block contained 20 stimuli, with the stimulus duration of 300 ms and the inter-stimulus interval of 500 ms. During the task block, participants were instructed to pay attention to the stimuli and press the button when they noticed a stimulus appearing twice non-intermittently (a one-back task). The order of different kinds of blocks was counterbalanced within and across scans. Participants performed three localizer scans for a total of 6 blocks of each type, lasting for 7 min.

### fMRI Data Acquisition and Preprocessing

Scanning was performed using a 3T Siemens MAGNETOM Skyra MRI system (Erlangen, Germany) with a whole head coil. A high-resolution 3D T1-weighted MRI scan was acquired using a magnetization-prepared rapid gradient-echo sequence. Functional images were obtained using a gradient-echo planar imaging sequence (TR 2500 ms, TE 30 ms, flip angle 75°, FOV 220 mm, matrix size 64 × 64, in plane resolution 3.5 × 3.5 mm). Each functional volume consisted of 45 axial slices of 3.4 mm with no inter-slice gap and covered the whole cerebrum and cerebellum.

Preprocessing of the imaging data as performed in FSL, consisted of brain extraction, slice timing correction, motion correction, and spatial smoothing (6 mm FWHM Gaussian kernel). Differently, the functional data of the localizer task remained in subject-specific space for the definition of ROIs (FFA and PPA). The functional images of the WM task were registered to the individual’s structural scan and the MNI152 standard space template with a 2 mm resolution using FMRIB’s Linear Image Registration Tool (FLIRT). Low frequencies (cutoff 128 s) were removed from the functional data of two tasks by a high-pass filter.

### Analysis of Event-Related fMRI Experiment

The WM task was a 2 × 2 experimental design, with two factors of cue type (face or scene) and target category (face or scene). There were four conditions of trials (Fcue_Ftarget denoting the cue type of face and target category of face, Scue_Ftarget, Fcue_Starget, and Scue_Starget). For the analysis of behavioral data, WM performance was evaluated by accuracy and response time (RT) of those trials in which participants made correct answers in Task 1. Statistical significance of behavioral differences was separately assessed on accuracy and RT, using repeated-measures ANOVAs and paired two-tailed *t*-tests.

For individual analyses of fMRI data, cue-related activity was identified by convolving a vector of maintenance period (from the onset of the cue to the onset of the probe stimulus) with the canonical synthetic hemodynamic response function (HRF) and its temporal derivative. The general linear model (GLM) as performed in FSL, was used to model the effects including main effect of two factors, interaction effect and pairwise effects (Fcue_Ftarget > Scue_Ftarget and Scue_Starget > Fcue_Starget). Motion parameters were included in the GLM to account for motion-related variance. Group analyses were conducted on Montreal Neurological Institute (MNI) normalized data, using random effects model to assess each effect. Statistical threshold was set at *Z* > 2.3 and *p* < 0.05, FDR corrected at cluster level.

### ROI-Based Univariate Analysis

According to the localizer task, the FFA of each participant was defined as the activated area in the fusiform gyrus for the contrast of viewing faces > viewing scenes (*p* < 10^−4^; Kanwisher et al., 1997). And the PPA was identified as the activated region in the posterior parahippocampal cortex for the contrast of viewing scenes > viewing faces (*p* < 10^−4^; Epstein and Kanwisher, 1998). Given that the right FFA and left PPA have been shown to be more strongly activated by faces and scenes, respectively (Kanwisher et al., 1997), they were chosen as the ROIs for all participants.

The main trial stages were modeled as stick functions (events) convolved with the canonical HRF in a GLM by FSL. The onset of the maintenance regressor was time-locked with the cue onset, and the onset of the probe regressor was time-locked with target-stimulus onset. Moreover, motion parameters were considered as the covariates in the GLM. The regressor vector eventually resulted in scalar β weights, measuring the relative changes of signal strength during each trial stage. The mean β values of the maintenance stage were calculated in each ROI (FFA and PPA) across the trials of each condition (Fcue_Ftarget, Scue_Ftarget, Fcue_Starget and Scue_Starget). Group differences of univariate effects were evaluated using paired two-tailed *t*-tests with *p* < 0.05 for statistical significance.

### Functional Connectivity

ROI-based functional connectivity maps of the whole brain were estimated for each participant, as described previously using a β-series correlation analysis approach (Gazzaley et al., 2004; Rissman et al., 2004). Mean β values of each ROI (FFA, PPA) were correlated with every brain voxel in subject’s native space for each participant and each condition (Fcue_Ftarget, Scue_Ftarget, Fcue_Starget and Scue_Starget). Single-participant functional connectivity maps were then normalized to the standardized MNI space and spatially smoothed (6 mm FWHM Gaussian kernel) for group analysis. Non-parametric permutation tests were performed to estimate whole-brain contrast maps between the conditions of Fcue_Ftarget and Scue_Ftarget for the ROI of FFA, and between the conditions of Scue_Starget and Fcue_Starget for the ROI of PPA. Statistical threshold was set at *p* < 0.01, FDR corrected at cluster level.

### Neurobehavioral Correlation Analysis

Correlation analysis was performed between functional connectivity differences (Fcue_Ftarget – Scue_Ftarget) based on the ROI of FFA and the WM performance improvements (Fcue_Ftarget – Scue_Ftarget; for accuracy and RT, respectively), and between functional connectivity differences (Scue_Starget – Fcue_Starget) based on the ROI of PPA and the WM performance improvements (Scue_Starget – Fcue_Starget). Statistical threshold of neurobehavioral correlations was set at *p* < 0.05, after Bonferroni correction for multiple comparisons. Furthermore, the Pearson–Filon statistic based on Fisher’s r-to-Z transformation (ZPF) was used to compare these two kinds of neurobehavioral correlations based on FFA and PPA, respectively (Raghunathan et al., 1996).

## Results

### Behavioral Performance

**FIGURE 2:**
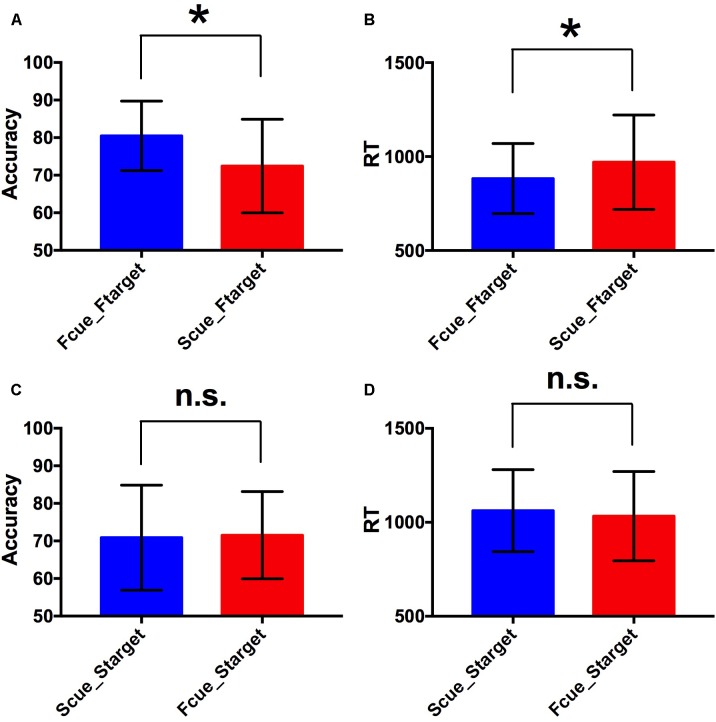
Behavioral performance. **(A,B)** Comparisons of WM accuracy and RT between Fcue_Ftarget and Scue_Ftarget. By paired two-tailed *t*-tests, if the target was a face, the accuracy in Fcue_Ftarget was significantly higher and RT was significantly shorter, compared to the corresponding values in Scue_Ftarget (*p* = 0.003 and 0.005, respectively). **(C,D)** Comparisons of WM accuracy and RT between Fcue_Starget and Scue_Starget. If the target was a scene, there were no significant differences of accuracy or RT between groups of different cue types (both *p*-values > 0.1). The symbols (^∗^) and (n.s.) indicate being and not being significant, respectively.

For the two factors of cue type (face or scene) and target category (face or scene) in WM task, a 2 × 2 repeated-measures ANOVA was separately performed on WM accuracy and RT. There was statistical significance of interaction effect and main effect of two factors for WM accuracy [*F*_(1,17)_ = 9.81, *p* < 0.005 for interaction effect; *F*_(1,17)_ = 4.71, *p* < 0.05 for main effect of cue type; *F*_(1,17)_ = 5.78, *p* < 0.05 for main effect of target category]. And there was statistical significance of interaction effect and main effect of target category for RT [*F*_(1,17)_ = 16.9, *p* < 0.001 for interaction effect; *F*_(1,17)_ = 2.66, *p* > 0.1 for main effect of cue type; *F*_(1,17)_ = 28.36, *p* < 0.001 for main effect of target category]. By paired two-tailed *t*-tests, if the target was a face, the accuracy in Fcue_Ftarget was significantly higher and RT was significantly shorter, compared to the corresponding values in Scue_Ftarget (*p* = 0.003 and 0.005, respectively; Figure 2A,B). However, if the target was a scene, there were no significant differences of accuracy or RT between groups of different cue types (both *p*-values > 0.1; Figure 2C,D). Therefore, only faces were recalled with improved WM performance due to the effect of incidental cue, which indicated that the incidental cue could bring faces into a privileged WM state during WM maintenance period, but not scenes.

### fMRI Results

**FIGURE 3:**
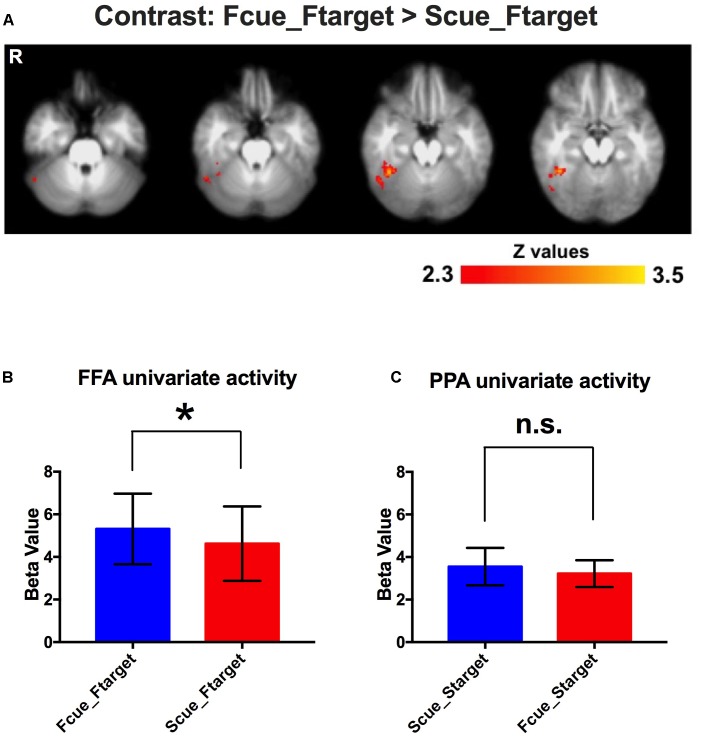
Event-related fMRI results. **(A)** A significantly activated region in the fusiform gyrus for the contrast of Fcue_Ftarget > Scue_Ftarget. Statistical threshold was set at *Z* > 2.3 and *p* < 0.05, FDR corrected at cluster level. **(B,C)** Univariate activities of FFA and PPA in different conditions. Univariate FFA activity during WM maintenance period was increased when the cue pointing to a face, compared to that when the cue pointing to a scene (Fcue_Ftarget > Scue_Ftarget; *p* = 0.022). For univariate PPA activity, there was no significant difference between groups of Scue_Starget and Fcue_Starget (*p* = 0.16). The symbols (^∗^) and (n.s.) indicate being and not being significant, respectively.

To investigate neural underpinnings of the incidental cue’s effect, the conventional 2-stage random effects model was performed for fMRI analysis. A significantly activated region in the fusiform gyrus was identified for the contrast of Fcue_Ftarget > Scue_Ftarget (Figure 3A). And this region was completed covered by the significant activations for interaction effect (Supplementary Figure S1). Moreover, no activation was observed in the posterior parahippocampal cortex for the contrast of Scue_Starget > Fcue_Starget, which was consistent with the behavioral results. To confirm whether the significantly activated region in the fusiform gyrus was in FFA, a ROI-based univariate analysis was performed to investigate cue-driven baseline activity shifts in FFA. As we expected, univariate FFA activity during WM maintenance period was increased when the cue pointing to a face, compared to that when the cue pointing to a scene (Fcue_Ftarget > Scue_Ftarget; *p* = 0.022; Figure 3B). The similar analysis, based on PPA, failed to identify a significant difference between groups of Scue_Starget and Fcue_Starget (Scue_Starget > Fcue_Starget; *p* = 0.16; Figure 3C). These results indicate that neural representations of the cue effect are dependent on the category of stimuli, comparable with the WM performance discrepancy.

### Function Connectivity Results

**FIGURE 4:**
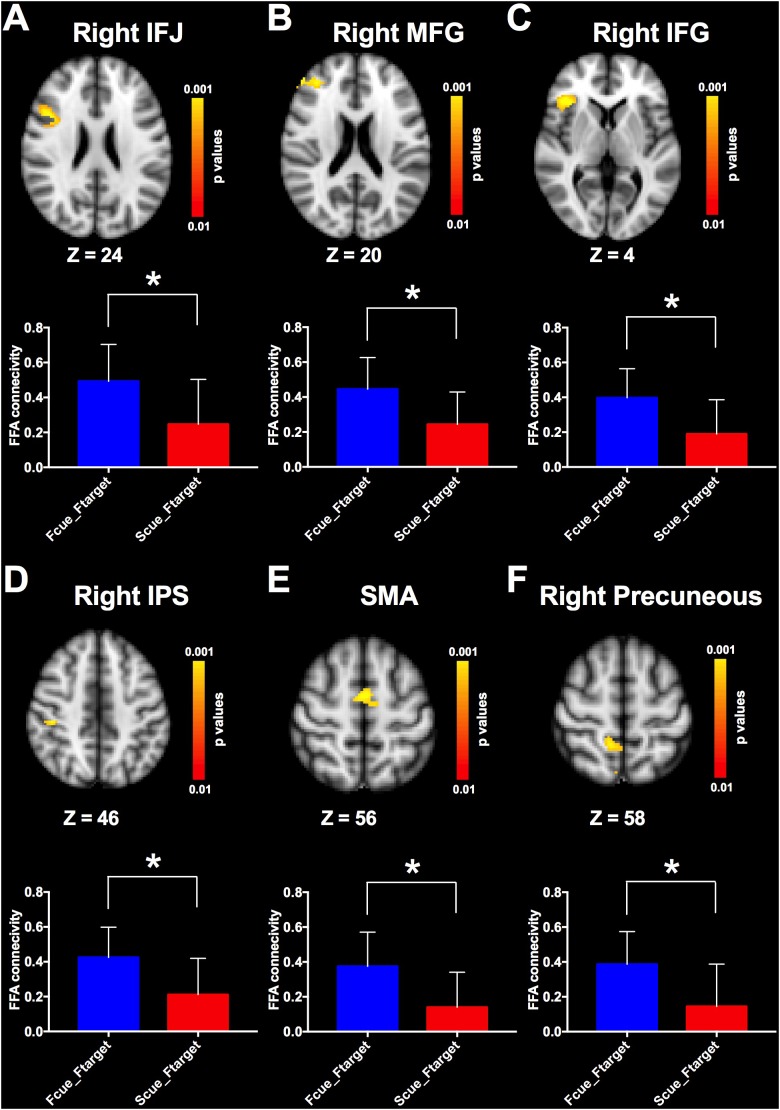
Comparisons of FFA functional connectivity for the contrast of Fcue_Ftarget > Scue_Ftarget. The FFA connectivity with right IFJ **(A)**, right MFG **(B)**, right IFG **(C)**, right IPS **(D)**, SMA **(E)**, and right precuneus **(F)** were significantly increased in Fcue_Ftarget, in contrast to that in Scue_Ftarget. Statistical threshold was set at *p* < 0.01, FDR corrected at cluster level. Stereotaxic MNI coordinated and mean *p*-values for significant regions are shown in Table 1. The symbol (^∗^) indicates being significant.

Given that cue-driven memory benefits and baseline activity shifts were found only on faces and FFA, respectively, the ROI-based functional connectivity analysis focused on the trials in which the target was a face. FFA connectivity maps of the whole brain were estimated by the β-series correlation method for each participant. A non-parametric analysis was used to compare FFA connectivity maps during WM maintenance period of different cue types. The FFA connectivity with right IFJ, MFG, IFG, right IPS, supplementary motor area (SMA) and right precuneus were significantly increased in Fcue_Ftarget, in contrast to that in Scue_Ftarget (Figure 4 and Table 1), which suggested these frontal and parietal regions might be engaged in potential top-down modulation of FFA activity.

**Table 1:** FFA connectivity comparisons (contrast: Fcue_Ftarget > Scue_Ftarget).

Brain region	No. voxels	Mean *p*-value	MNI_X	MNI_Y	MNI_Z
R inferior frontal gyrus	78	0.004	38	26	6
L inferior frontal gyrus	43	0.005	−32	24	8
R middle frontal gyrus	216	0.004	−34	42	12
L middle frontal gyrus	84	0.004	36	42	20
R inferior frontal junction	233	0.003	44	14	24
R intraparietal sulcus	56	0.004	42	−28	46
R superior parietal lobule	38	0.007	34	−36	44
R precuneus	82	0.006	10	−46	58
Supplementary motor area	115	0.004	0	−4	56

*L, Left; R, Right; MNI_X, Coordinate X in MNI space; MNI_Y, Coordinate Y in MNI space; MNI_Z, Coordinate Z in MNI space.*

### Neurobehavioral Correlations

**FIGURE 5:**
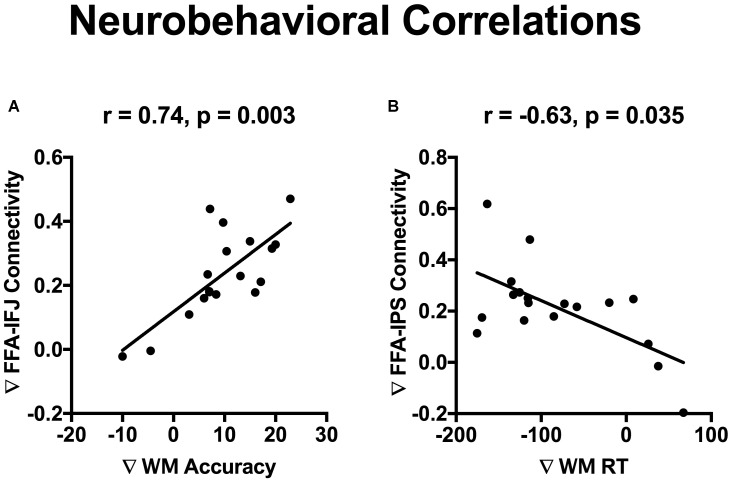
Neurobehavioral correlations. Significant correlations were revealed between WM accuracy increase and connectivity differences of FFA-IFJ (*r* = 0.74; *p* = 0.003; **A**), and between WM RT decrease and connectivity difference of FFA-IPS (*r* = –0.63; *p* = 0.035; **B**), after Bonferroni correction for multiple comparisons.

The incidental cue brought faces into a privileged state and resulted in a benefit on WM performance. To investigate whether cue-driven FFA connectivity changes were associated with the behavioral benefit, correlation analysis was conducted between differences of FFA connectivity with those frontal and parietal regions (Fcue_Ftarget – Scue_Ftarget), and the WM improvements (Fcue_Ftarget – Scue_Ftarget; for accuracy and RT, respectively). Significant correlations were revealed between accuracy increase and connectivity differences of FFA-IFJ (*r* = 0.74; *p* = 0.003; Figure 5A), and between RT decrease and connectivity difference of FFA-IPS (*r* = −0.63; *p* = 0.035; Figure 5B), after Bonferroni correction for multiple comparisons. All the results of neurobehavioral correlations were shown in Supplementary Figure S2. What’s more, by the Pearson-Filon statistic, the correlation between accuracy increase and connectivity differences of FFA-IFJ was significantly higher than the correlation between accuracy increase and connectivity differences of PPA-IFJ (ZPF = 2.01, *p* = 0.022, one-tailed), and the same result was obtained for the correlation between RT decrease and connectivity difference of FFA-IPS (ZPF = 1.84, *p* = 0.033, one-tailed). These results suggested that top-down modulation might be involved in cued-driven WM benefits for faces, but not scenes.

## Discussion

By the behavioral paradigm of incidental cue, our study provides initial evidence that there is a privileged WM state on complex objects of faces, but not scenes during WM maintenance period. fMRI analysis revealed an activated brain region in FFA underlying memory benefits of faces, which was potentially modulated by a frontoparietal network of regions (right IFJ, MFG, IFG, right IPS, right precuneus and SMA) via their functional couplings with FFA. Furthermore, FFA connectivity with IFJ and IPS could predict cue-driven improvements of WM performance.

The effect of the incidental cue during WM maintenance period was first found in orientation-discriminating WM task, which provided direct evidence for the existence of at least two different representational states of WM (Zokaei et al., 2013, 2014). The incidental cue, different from the valid retro-cue, was completely uninformative about which object or which category of objects would be probed. For complex objects such as faces and natural scenes, the incidental cue behaved variously for different categories of stimuli. In this study, we revealed that the incidental cue could bring faces into a privileged WM state, but not scenes. The similar phenomenon was found in the effect of the category-predictive cue during WM expectation period, which only worked for faces, not scenes (Bollinger et al., 2010). One explanation in the paper was that the diversity of scenes features makes it difficult to generate a robust template and faces are more stereotyped than scenes. We provided another explanation that faces, as the most important and salient visual stimuli a human encounters, are a special kind of objects, which are thought to be processed and represented in a holistic manner (Tanaka and Farah, 2003). In our situation, the holistic representational state of faces in WM possibly made them easy to be prioritized by the incidental cue, just as the basic feature of orientation. And our results indicate that the incidental cue during WM maintenance period enhances memory of both low-level elementary features and high-level complex faces.

In current study, we could measure the WM performance of both cued and uncued stimuli with the behavioral paradigm of incidental cue. In addition, we could distinguish and determine category-selective neural underpinnings of the incidental cue’s effect for the contrast of cued > uncued stimuli, using two categories of stimuli (faces and scenes) in the event-related fMRI experiment. As we expected, we found consistent results in behavioral performance and fMRI analysis. There were significant differences of WM accuracy and RT, between cued faces and uncued faces. Meanwhile, A significantly activated region in the fusiform gyrus was revealed for the contrast of cued > uncued faces, and cue-driven baseline activity shifts were identified in the face-selective region of FFA. However, all these corresponding results failed to be found on scenes and the scene-selective region of PPA. Besides, the neurobehavioral correlations based on FFA were significantly higher than those based on PPA. Thus, as the complex objects, faces and natural scenes are not only processed in different brain regions, but also possibly stored in WM of different patterns.

Different representational states of faces in WM were accompanied with sensory activity biasing in FFA, which was potentially mediated via top-down control from a frontoparietal network. Our study proved that FFA connectivity with right IFJ, MFG, IFG, right IPS, SMA and right precuneus were significantly increased for cued faces during WM maintenance period, in contrast to that of uncued faces. What’s more, FFA connectivity with IFJ and IPS could predict WM improvements. The top-down modulation involved with a frontoparietal network of regions was studied not only during WM maintenance period, but also in other WM periods (Gazzaley and Nobre, 2012). During WM expectation period, Bollinger et al. (2010) found the effect of predictive category cueing for faces in an object delayed-response task and the degree of functional connectivity between FFA and brain regions of prefrontal and parietal cortex (right IFJ, MFG, IFG, and IPS) correlated with the magnitude of pre-stimulus activity modulation in FFA. Particularly, IFJ, defined by Brass and von Cramon (2004), is located at the intersection of the inferior frontal sulcus and precentral sulcus. It’s proved to be a functionally discrete region and a key node connecting the dorsal and the ventral attention networks (Szczepanski et al., 2010). During WM encoding period, a fMRI study revealed visual cortical areas that selectively processing relevant information were functionally connected with the frontal-parietal network including IPS, IFJ and MFG, while those processing irrelevant information were coupled with the default network (Chadick and Gazzaley, 2011). Interestingly, the degree of coupling between visual cortices and default network regions predicted the WM performance. During WM maintenance period, Kuo et al. (2018) found that the retro-cues modulated the strength of functional connectivity between the frontoparietal and early visual areas in favor of the most relevant information. Previous studies and our findings indicated top-down modulation might play an important part in multiple stages of representations supporting WM performance.

About neural circuit mechanisms of WM, recent neurophysiological studies revealed stable population coding within a specific subspace coexisting with heterogeneous neural dynamics in prefrontal cortex during WM maintenance period (Murray et al., 2017). Our finding about the coexistence of at least two different representational states of WM was possibly explained by neural population coding within different subspaces. However, it’s unclear whether the incidental cue, similar to an uninformative retro-cue, has an effect to strengthen the WM representation of cued item or inhibit the WM representation of uncued item to protect the selected representation from interference (Souza and Oberauer, 2016). Bays and Taylor (2018) presented a population coding model suggesting retro-cue can’t increase the total information stored about a stimulus and protects items from time-based decay instead, which are supportive for the latter explanation. In this study, for different categories of complex objects, the privileged WM state was only found for faces due to the incidental cue, not natural scenes, which suggested different patterns of neural population coding for faces and scenes, with different responses to the incidental cue. Thus, this kind of behavioral discrepancy could have some implications for neural circuit mechanisms and proper computational models of WM.

## Conclusion

In conclusion, under the effect of the incidental cue, the privileged WM state and potential top-down modulation existed for faces, but not scenes, during WM maintenance period.

## Ethics Statement

This study was carried out in accordance with the recommendations of the ethics committee in University of Helsinki with written informed consent from all subjects. All subjects gave written informed consent in accordance with the Declaration of Helsinki. The protocol was approved by the ethics committee in University of Helsinki.

## Author Contributions

HL and SC contributed conception and design of the study. HL organized the database, performed the statistical analysis, and wrote the first draft of the manuscript. All authors contributed to manuscript revision, read, and approved the submitted version.

## Conflict of Interest Statement

The authors declare that the research was conducted in the absence of any commercial or financial relationships that could be construed as a potential conflict of interest.

## Supplementary Material

FIGURE S1 The cue × target interaction effect.

FIGURE S2 All Neurobehavioral Correlations.
