# Supplementary figures and images for: A Privileged Working Memory State and Potential Top-Down Modulation for Faces, Not Scenes
Source: Front Hum Neurosci. 2019 Jan 28;13:2. doi: 10.3389/fnhum.2019.00002 (PMC6360155; doi:10.3389/fnhum.2019.00002)

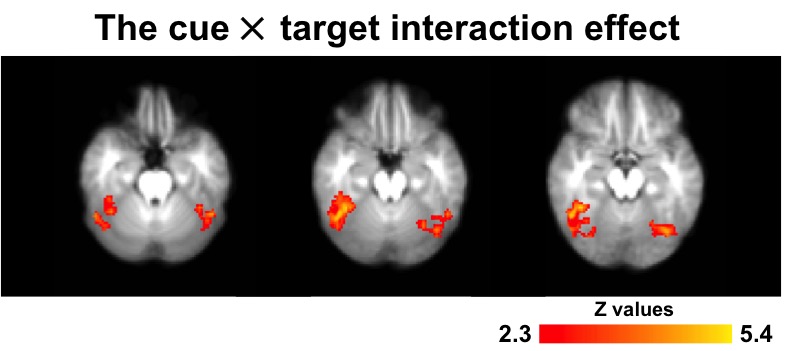

Supplement: FIGURE S1 — The cue × target interaction effect. [file Image_1.JPEG]

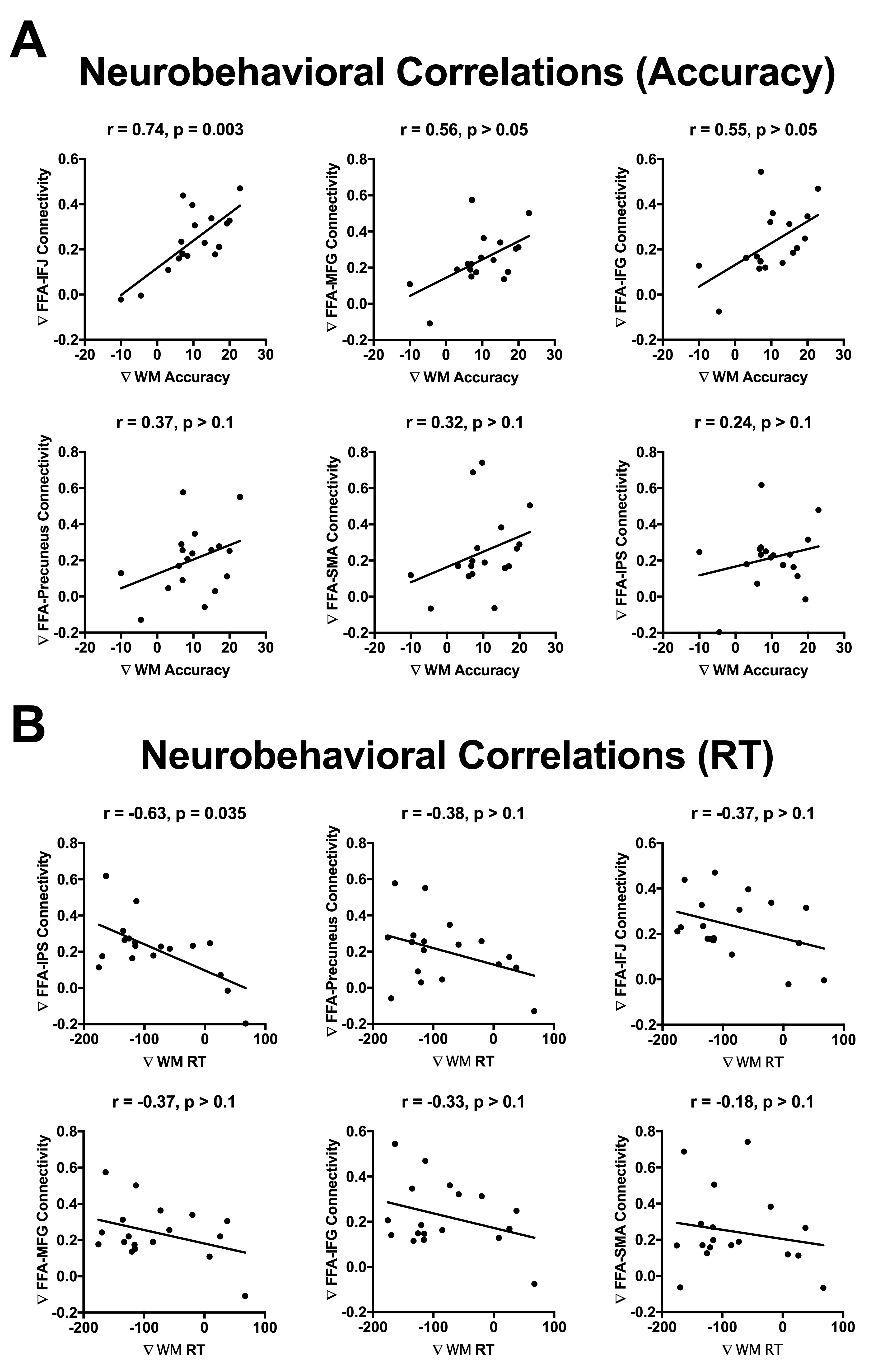

Supplement: FIGURE S2 — All Neurobehavioral Correlations. [file Image_2.JPEG]
